# Clinical observation of umbilical cord mesenchymal stem cell transplantation in treatment for sequelae of thoracolumbar spinal cord injury

**DOI:** 10.1186/s12967-014-0253-7

**Published:** 2014-09-12

**Authors:** Hongbin Cheng, Xuebin Liu, Rongrong Hua, Guanghui Dai, Xiaodong Wang, Jianhua Gao, Yihua An

**Affiliations:** Department of Cell Transplantation, General Hospital of Chinese people’s Armed Police Forces, Beijing, 100039 China; Department of CT Room Radiology, General Hospital of Chinese people’s Armed Police Forces, Beijing, 100039 China

**Keywords:** Umbilical cord mesenchymal stem cell, Spinal cord injury, Transplantation, Urodynamic examination

## Abstract

**Background:**

Umbilical cord mesenchymal stem cells (UCMSCs) have a considerable advantage and potential in treating for central nervous system diseases and have become a novel alternative treatment for spinal cord injury. This study aims to compare the neurological function outcome of stem cell transplantation, rehabilitation therapy, and self-healing for sequelae of spinal cord injury.

**Methods:**

Thirty-four cases of thoracolumbar spinal cord injury were randomly divided into three groups: the stem cell transplantation group was given CT-guided UCMSC transplantation twice; the rehabilitation group received rehabilitation therapy; and the blank control group did not receive any specific treatment. AIS grading, ASIA scoring, the manual muscle strength and muscle tension scale, and the Barthel index were used to evaluate the clinical outcome. Urodynamic examination was also performed for patients in the UCMSC group and the rehabilitation therapy group.

**Results:**

Seven of the ten patients in the UCMSC group had significant and stable improvement in movement, self-care ability, and muscular tension; five of the forteen patients (36%) in the rehabilitation group also had certain improvement in these aspects. Urodynamic examination demonstrated that patients in the UCMSC group exhibited an increase in maximum urinary flow rate and maximum bladder capacity, as well as a decrease in residue urine volume and maximum detrusor pressure. The rehabilitation group exhibited decreased maximum bladder capacity, but no perceptible change in maximum urinary flow rate, residue urine volume or maximum detrusor pressure.

**Conclusions:**

UCMSC transplantation can effectively improve neurological functional recovery after spinal cord injury, and its efficacy is superior to that of rehabilitation therapy and self-healing.

**Trial registration:**

The present clinical study was registered at chictr.org (registration number: NCT01393977).

## Background

Thoracolumbar spinal cord injury has a relatively high rate of incidence, and reports have indicated that cervical, thoracic and lumbar spine injuries account for 4.9%, 28.0%, and 65.9% of total thoracolumbar spinal injuries, respectively [1]. Thoracolumbar spinal cord injuries are manifested primarily by paralysis of the lower limbs, bowel and bladder dysfunction, and sympathetic nervous system disorder [2,3]. Neuroprotective treatment can be performed in acute phases with ganglioside (GM-1), mouse nerve growth factor (NGF), etc. [4,5]. Conventional rehabilitation therapy was the preferred treatment for convalescence and sequelae-phase spinal cord injury. Occupational therapy, limb massage, functional breathing and defecation training, etc. can to some extent delay disuse muscle atrophy and retain a portion of limb function [6,7].

Axon fractionation and extensive demyelination after spinal cord injury may impede neural regeneration. Tthrombosis, rupture and occlusion of blood vessels supporting the spinal cord may lead to ischemia and hypoxia; micro-environment changes at the damage site can cause massive inflammatory cell infiltration and inflammatory cytokine release that also impede neural regeneration. Bleeding, inflammatory factor secretion, and arachnoid rupture after injury may result in disorders in local cerebrospinal fluid circulation that lead to aggregation of toxic substances and blockage of neurotrophic factors transporting to the site of injury. A local glial scar may form after injury, which impedes axon growth [8-11]. These factors represent the obstacles in neurofunctional recovery that cannot be overcome by conventional rehabilitation therapy.

In recent years, stem cell transplantation has become a novel alternative treatment for sequelae of spinal cord injury, and a large number of reports have covered this topic. In a canine model, neural stem cells transplanted at the site of a hemitransected spinal cord can differentiate into mature neurons and oligodendrocytes, promoting recovery of the spinal cord function [12]. Canine bone marrow mesenchymal stem cells transplanted into the site of spinal cord injury promoted nerve cell regeneration and reduced fibrosis, thereby contributing to the recovery of neural function [13]. Adipose tissue-derived mesenchymal stem cells (ADMSCs) can be used in the treatment for sequelae of spinal cord injury more than 1 year after initial injury, and is proved to be safe in the follow-up [14]. Transplantation of human umbilical cord blood-derived mesenchymal cells into rats at the hemitransected spinal cord showed significant improvement in electrophysiological examination [15]. In the treatment of acute spinal cord injury, human umbilical cord mesenchymal cells transplanted into rats promoted the recovery of spinal cord morphology and function [16]. Umbilical cord mesenchymal stem cell (UCMSCs) can differentiate into neurons, glial cells and vascular endothelial cells under specific inducing conditions. UCMSCs can also generate cytokines and neurotrophic factors including vascular endothelial growth factor (VEGF), glial cell line-derived neurotrophic factor (GDNF) and brain derived neurotrophic factor (BDNF) that support neural regeneration, promote axon growth, and activate damaged neurons. In addition, UCMSCs can also inhibit glial scar formation, alleviate scar obstructions, and activate endogenous neural stem cells [17-21]. This type of stem cell is widely available without any ethical conflicts or damage to the cell donor and thus has great advantage and potential in treatment for central nervous system diseases.

This study used umbilical cord mesenchymal stem cells for transplantation and compared neurofunctional outcomes of patients suffering sequelae of thoracolumbar spinal cord injury that were treated with stem cell transplantation, rehabilitation training, or no treatment.

## Methods

### Study subjects

As shown in Table 1, 34 cases of thoracolumbar spinal cord injury that were graded ‘A’ by the AIS grading system were randomly divided into 3 groups: the stem cell transplantation group, the rehabilitation therapy group, and the blank control group. The 34 patients were diagnosed by magnetic resonance (MR) and computed tomography (CT) examination 12–72 (23.19 ± 15.67) months post-injury, were aged 19–57 (35.25 ± 8.96) years and were injured at a point between the 10th thoracic vertebra (T10) to the 1st lumbar vertebra (L1). The injuries were caused by a traffic accident in 16 cases, falling from high place in 7 cases, heavy pound in 5 cases, and a sword wound in 6 cases. Excluding criteria were as follows: 1. anemia, hypoalbuminemia, or diabetes; 2. syringomyelia, spinal cord compression, or tethered cord; 3. pressure ulcers or infectious diseases (such as pneumonia, bacteremia, sepsis, etc.); 4. severe heart, pulmonary dysfunction; 5. highly allergic constitution; and 6. renal pelvis and ureter hydronephrosis or urinary tract stones that lead to renal insufficiency. The three groups showed no significant difference in age or post-injury age. Patients of the stem cell transplantation group all signed consent forms for stem cell therapy. The entire treatment protocol is approved by the Ethics Committee of the General Hospital of Chinese People's Armed Police Forces.

**Table 1:** **General information of the patients in the three groups**

	**Average age (years)**	**Time past injury (months) [min,max]**		**AIS grading**
UCMSC group	35.30 ± 8.23	21.40 ± 12.96	[12,50]	A
Rehabilitation group	36.64 ± 9.90	18.57 ± 11.35	[12,55]	A
Blank control group	32.40 ± 7.72	30.70 ± 19.99	[12,52]	A

### Cell preparation

Cell Preparation were performed as previously described [22]. Briefly, umbilical cord tissue was obtained from full-term healthy newborns after their parents signed the informed consent. The tissue was disinfected in 75% alcohol for 30 seconds, had the Wharton's jelly peeled off, and was then cut into 0.5 cm3 pieces. The sample was centrifuged at 250xg for 5 minutes and was cultured with α-MEM medium that contained 10% fetal bovine serum at 37°C in an incubator containing 5% CO_2_. The medium was changed every three days. Approximately 10 days later, the umbilical cord tissue was removed, and the cells that had attached to the plate were further cultured to 80% confluence and were passaged after digestion with 0.25% trypsin. Cells of the 6-8th passage were identified by flow cytometry to confirm their purity and to exclude bacteria, fungi and mycoplasma contamination, and were then used for transplantation. Fifty microliterss of cell suspension (cell concentration was 4 × 10^5^ cells/μl) was injected into two sites of the spinal cord, 25 μl at each site. The transplantation was repeated once. In total, 4 × 10^7^ cells were transplanted.

### Identification of UCMSCs

**Figure 1:**
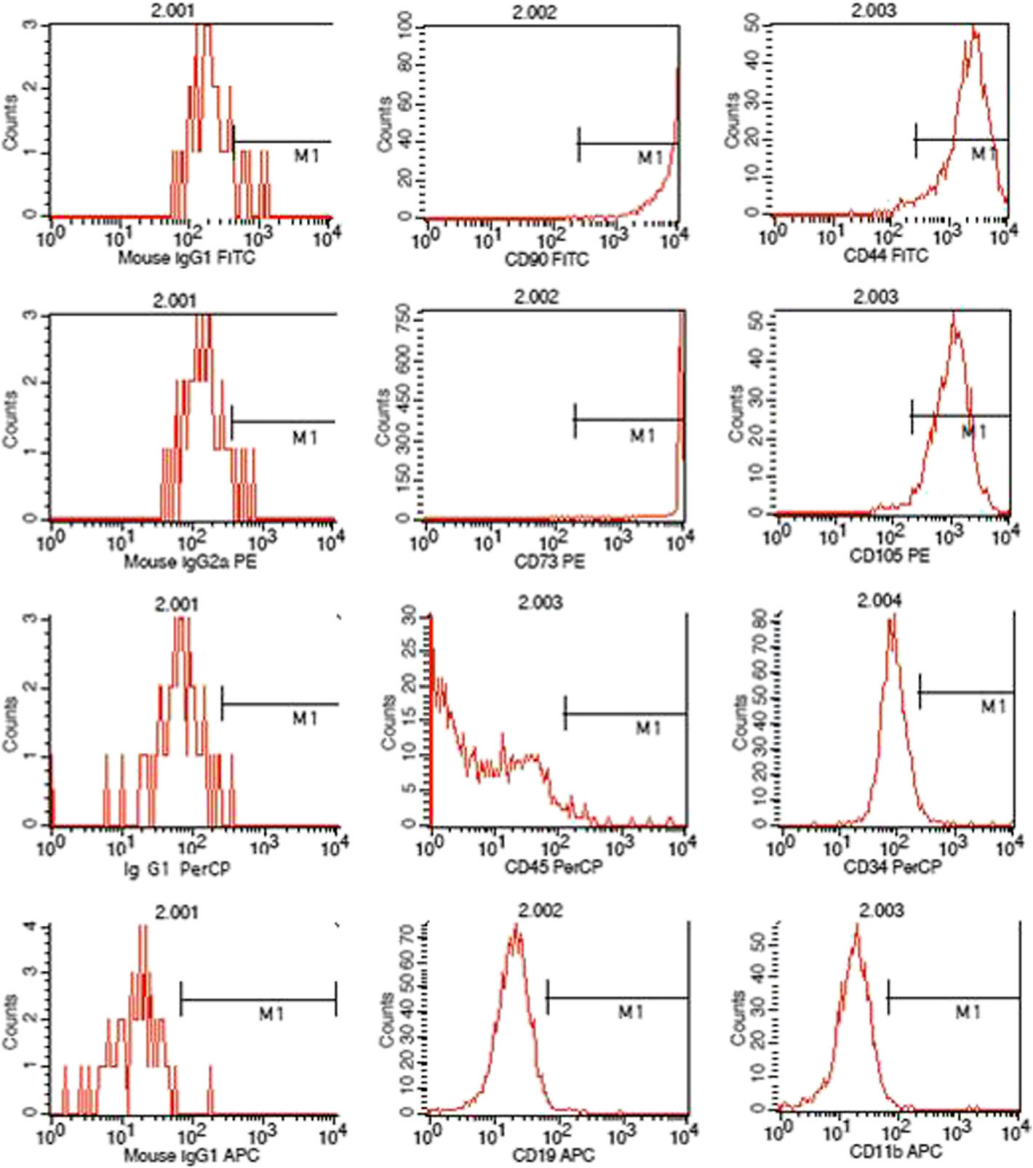
**Flow cytometry analysis for UCMSCs.** The levels of CD105, CD90, CD73 and CD44 were higher than 95%, whereas CD19, CD45, CD11b or CD34 were lower than 5%.

The flow cytometry was performed to detect the cell purity. Levels of CD105, CD90, CD73 and CD44 were all higher than 95%, whereas CD19, CD45, CD11b or CD34 were all lower than 5% (Figure 1). The cells were then used for transplantation.

### Therapeutic schedule

The schedule for stem cell transplantation group was as follows: patients first underwent neurological assessment and urodynamic examination and then received CT-guided spinal cord stem cell transplantation twice. The two transplantations were separated by 10 days. The patients received no further treatment of any type after transplantation and underwent neurological assessment and urodynamic examination again 6 months later.

**Figure 2:**
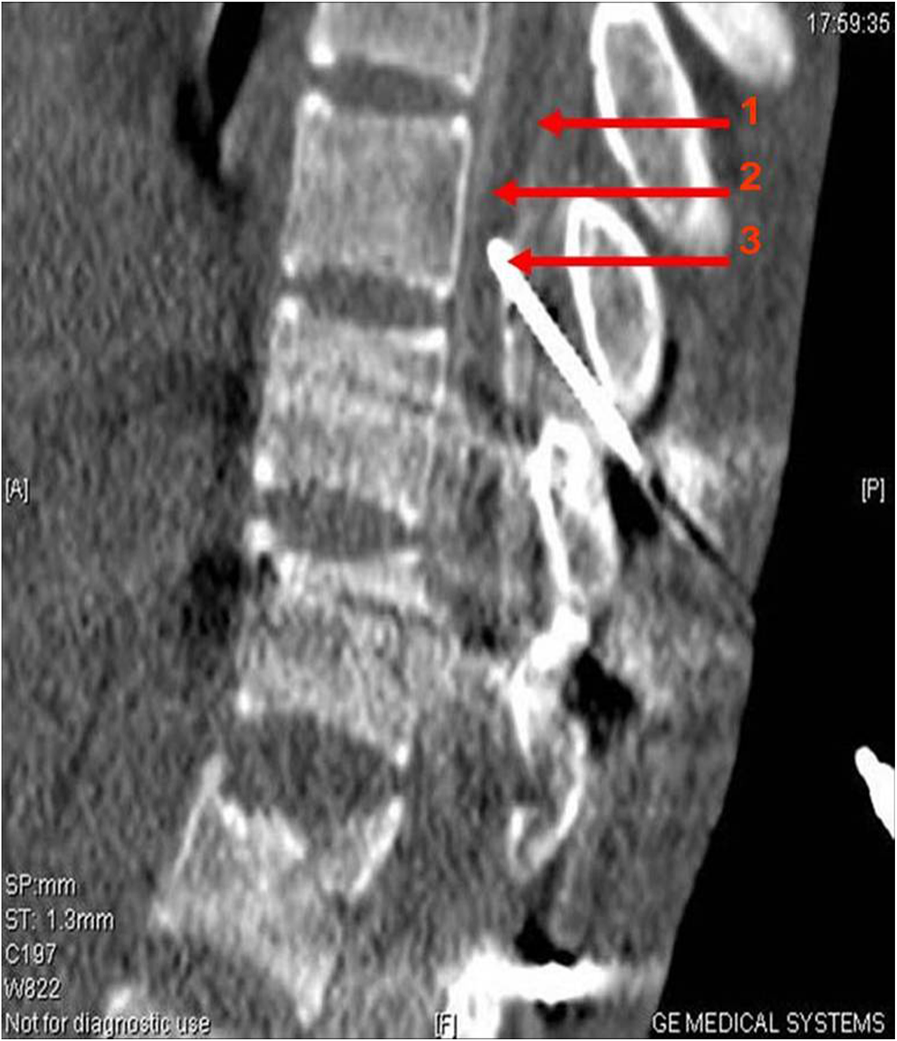
**A sagittal image: the needle reached the spinal cord.** The red line 1 indicates the subarachnoid; the red line 2 indicates the spinal cord; the red line 3 indicates the needle.

**Figure 3:**
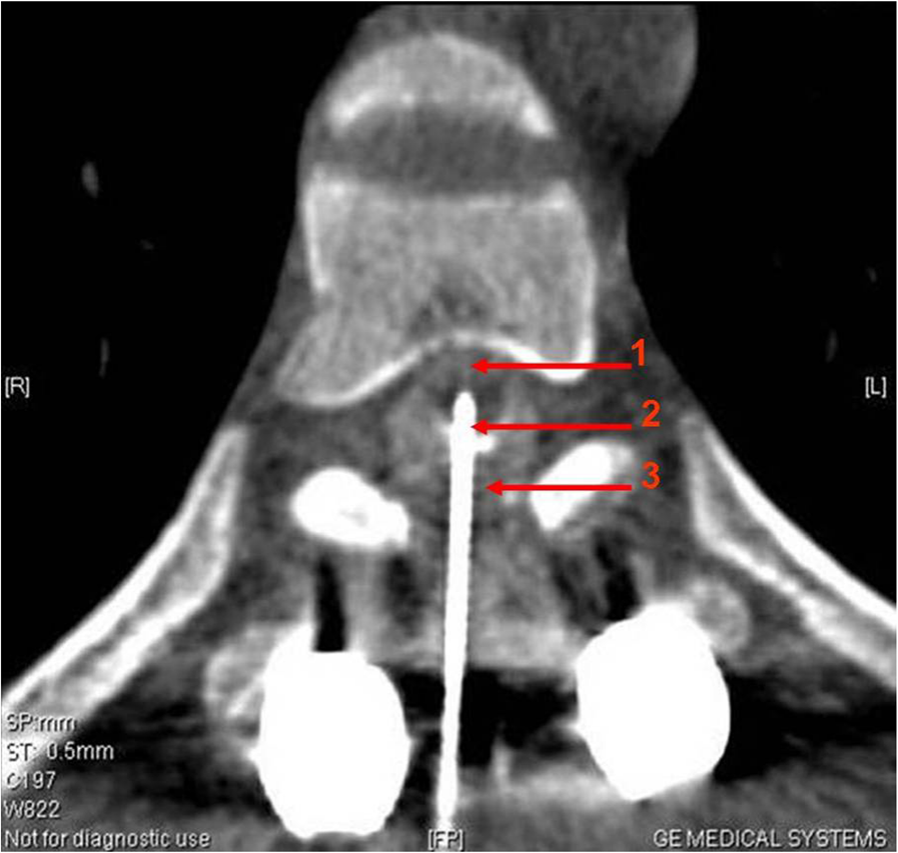
**An axial (transverse) image: the needle reached the spinal cord.** The red line 1 indicates the spinal cord; the red line 2 indicates the needle; the red line 3 indicates the subarachnoid.

Methods for CT-guided spinal cord stem cell transplantation were performed as previously described [22]. Briefly, the transplantation was performed in the CT room and a disposable 9# lumbar puncture needle was used. The patient lay in the prone position on the CT examination bed, according to an MR image and neurological examination. A puncture was made at the junction of the normal and injured spinal cord where stem cells were then transplanted. During the puncturing process, depth and direction of the puncture needle was adjusted according to the images of multiple CT scan and 3-dimensional reconstructions. When the needle penetrated into the subarachnoid space, iodixanol was used as a contrast medium to show the spinal cord (Figures 2 and 3). The needle was slowly punctured into the spinal cord, and a total of 2 × 10^7^ cells were injected in two aliquots. The two injections were spaced by 3 min, and the needle remained in place for another 3 min before being slowly withdrawn.

The treatment schedule for the rehabilitation therapy group was as follows: patients first underwent neurological assessment and urodynamic examination and then received rehabilitation therapy. The rehabilitation therapy included functional recovery training and urinary retention training. The treatment course lasted for 90 days with no further treatment of any type, and the patients were examined again 6 months later.

The treatment schedule for the blank control group was as follows: patients were left at home for self-healing with no specific treatment.

### Efficacy evaluation

All of the neurofunctional assessments were performed by a single rehabilitation assessor who had no knowledge of the grouping or treatment. Muscle strength, muscle tension, sensation of the paralyzed limbs, and self-care ability were evaluated according to the ASIA score, the manual muscle strength and muscle tension scale, and the Barthel Index. Patients of the stem cell group, rehabilitation group, and blank control group were examined before treatment and 6 months after treatment.

### Urodynamic examination

Bladder pressure, bladder volume, voiding pressure, and flow rate were tested using the Medtronic urodynamic system. Maximum urinary flow rate, maximum bladder capacity, residual urine volume, and maximum detrusor pressure were determined. The stem cell transplantation group and rehabilitation therapy group were examined prior to and 6 months post treatment.

### Statistical analysis

All of the data obtained were processed by SPSS13 software and are shown as the means ± standard deviations. Each neurofunctional score before and after treatment was compared using the paired t-test. P < 0.05 was regarded to be statistically significant.

## Results

### Assessment

After stem cell transplantation, the patients showed improvement in sensation; the score was raised from 146.60 ± 22.35 to 148.30 ± 20.99, but it was not statistically significant (Table 2, P > 0.05). Motion, muscle tension, and self-care ability significantly improved; that is, the strength of waist, abdomen, and lower limbs increased, excessive muscle tension decreased, and self-care ability was strengthened (Table 2, P < 0.05). The patients in the rehabilitation group improved in sensation, motion, and self-care ability, from 147.57 ± 29.63, 55.00 ± 6.66, and 37.86 ± 17.94 before treatment to 148.12 ± 23.33, 56.64 ± 9.09, and 38.21 ± 17.72 after treatment, respectively. However, the differences were not statistically significant (Table 3, P > 0.05). The muscle tension of this group increased from 14.29 ± 11.74 before treatment to 16.00 ± 13.34 after treatment, but the difference was also not statistically significant (Table 3, P > 0.05). The blank control group showed no significant changes in sensation, motion, muscle tension, or self-care ability (Table 4, P > 0.05).

**Table 2:** **Comparison of the assessments before and 6 months after stem cell treatment**

	**Sensation [min,max]**	**Motion [min,max]**	**Muscle tension [min,max]**	**Barthel index [min,max]**
Before treatment	146.60 ± 22.35 [104,168]	54.00 ± 11.48 [40,80]	9.90 ± 8.00 [0,20]	33.50 ± 6.69 [25,45]
After treatment	148.30 ± 20.99 [110,184]	58.00 ± 12.42 [45,89]	8.50 ± 7.43 [0,20]	41.40 ± 6.42 [30,50]
*P*	0.052	0.012	0.007	0.001

**Table 3:** **Comparison of the assessment before and 6 months after rehabilitation therapy**

	**Sensation [min,max]**	**Motion [min,max]**	**Muscle tension [min,max]**	**Barthel index [min,max]**
Before treatment	147.57 ± 29.63 [88,224]	55. 00 ± 6.66 [50,67]	14.29 ± 11.74 [0,38]	37.86 ± 17.94 [25,75]
After treatment	148.12 ± 23.33 [88,224]	56.64 ± 9.09 [50,74]	16.00 ± 13.34 [0,38]	38.21 ± 17.72 [10,75]
*P*	-	0.140	0.165	0.336

**Table 4:** **Comparison of the assessment before and 6 months later in control group**

	**Sensation [min,max]**	**Motion [min,max]**	**Muscle tension [min,max]**	**Barthel [min,max]**
Before treatment	140.60 ± 4.00 [136,144]	50.90 ± 0.23 [50,51]	3.40 ± 4.72 [0,10]	35.00 ± 8.17 [30,55]
After treatment	141.45 ± 3.12 [136,148]	52.56 ± 0.11 [50,51]	3.41 ± 3.62 [0,10]	33.16 ± 9.68 [35,60]
*P*	-	-	-	-

### Urodynamic analysis

All of the patients of the stem cell transplantation group underwent urodynamic examination before and after treatment. Six months after stem cell treatment, maximum bladder capacity significantly increased and maximum detrusor pressure significantly decreased compared with those variables before treatment (Table 5, P < 0.05). Although the maximum urinary flow rate seemed increase and the residual urine volume seemed to decrease, the differences were not statistically significant (Table 5, P > 0.05). Ten of the 14 patients of the rehabilitation group were subjected to urodynamic examination before and after rehabilitation therapy. All of the urodynamic indicators showed a tendency of deterioration, but none were statistically significant except for maximum bladder capacity (Table 6). Since the blank control group was not treated at all, urodynamic examination was not performed for this group to avoid complications related to this test (infection, urine leakage, hematuria, etc.).

**Table 5:** **Results of urodynamic analysis before and 6 months after stem cell treatment**

	**Maximum urinary flow rate (ml/s)**	**Maximum bladder capacity(ml)**	**Residual urine volume(ml)**	**Maximum detrusor** **pressure(cmH**_**2**_ **O)**
	**[min,max]**	**[min,max]**	**[min,max]**	**[min,max]**
Before transplantation	9.26 ± 11.74 [0,33.5]	389.50 ± 176.12 [140,600]	84.20 ± 94.60 [0,300]	60.00 ± 42.60 [0,120]
After transplantation	11.50 ± 17.50 [0,57.7]	505.30 ± 106.37 [322,655]	38.80 ± 51.49 [322,655]	33.14 ± 34.94 [0,98]
*P*	0.515	0.009	0.163	0.023

**Table 6:** **Results of urodynamic analysis before and 6 months after stem cell treatment**

	**Maximum urinary flow rate(ml/s)**	**Maximum bladder**	**Residual urine volume(ml)**	**Maximum detrusor** **pressure(cmH**_**2**_ **O)**
	**[min,max]**	**[min,max]**	**[min,max]**	**[min,max]**
Before transplantation	3.7 ± 5.12	433. 00 ± 41.17	54.00 ± 57.71	17.60 ± 39.35
	[0,10.3]	[400,500]	[0,150]	[0,88]
After transplantation	2.62 ± 3.90	397.00 ± 63.06	186.00 ± 115.02	19.00 ± 42.49
	[0,8.7]	[330,500]	[30,350]	[0,95]
*P*	0.222	0.043	0.082	0.374

### Radiation safety

CT-guided spinal cord stem cell transplantation was performed according to the European guidelines on quality criteria for computed tomography (European Community working paper-EUR16262.1997.4) and the European Radiological Protection Association. No serious radiation-related adverse events occurred during treatment or follow-up.

### Adverse reactions

Only one patient in the stem cell treatment group presented radiating neuralgia after surgery (10%), and this was alleviated spontaneously in 24 hr. The reason is that the injured spinal nerve formed the adhesions at different degrees after spinal cord injury. When the needle was slowly punctured into spinal cord, it directly simulted the nerve roots and caused the radiating neuralgia. No adverse effects were observed in other groups.

## Discussion

Through scaled ratings and urodynamic examinations, this study proved that transplantation of UCMSCs has advantages in neurofunctional recovery in comparison with rehabilitation therapy and self-healing alone. The stem cell transplantation group improved significantly in motor function (P = 0.012); that is, the muscle strength of the waist, abdomen, and lower limbs increased. Seven of the 10 patients had their muscle strength increased from level 0 to level 1 or 2 (data not shown), and motor function of the paralyzed limbs improved as muscle strength increased. The rehabilitation group and blank control group also showed some improvements but the difference was not statistically significant (P > 0.05). Regarding muscle tension, excessive muscle tension significantly decreased (P = 0.007) after stem cell transplantation. Eight patients showed excessive muscle tension before treatment, and 7 of them (87.5%) had their muscle tension decreased (data not shown); the rehabilitation group and blank control group showed no significant improvements in muscle tension (P > 0.05). As to self-care ability, the stem cell transplantation group significantly improved in activities such as bed-chair transfer, bowel and urinary retention, and ground movements, which are related to a decrease in excessive muscle tension and an improvement in movement ability of the paralyzed limbs. The rehabilitation group showed some improvements but the differences were not statistically significant (P > 0.05). The self-care ability of the blank control group decreased (P > 0.05), most likely representing functional decline of the limbs due to lack of treatment and use. All of the three groups improved in sensation, and the outcome of the stem cell transplantation group was superior to that of the other two groups, but the difference was not statistically significant. In addition, we observed that patients had their lower limb blood circulation improved after stem cell transplantation, which was manifested by an increase in skin temperature, change of skin color from pale to ruddy, a decrease in skin pigmentation, etc., but because no objective evaluation of these improvements is available, that was not quantified.

In summary, the efficacy of stem cell transplantation is superior to that of rehabilitation therapy and self-healing in the improvement of motor ability, muscle tension, self-care ability, etc. The rehabilitation group and blank control group showed difference primarily in self-care ability (the Barthel index of the rehabilitation group rose from 37.86 ± 17.94 to 38.21 ± 17.72, whereas that of the blank control group dropped from 35.00 ± 8.17 to 33.16 ± 9.68), and the patients of the rehabilitation group were likely taught some tricks in controlling their limbs and sphincters through bodily function recovery and urinary retention training and thus had an advantage over the blank control group. The blank control group showed no significant differences in sensation, movement, muscle tension, or self-care ability, which was in accordance with the conclusion that spinal cord function tends to be stable one year after injury [23], which is also why we chose patients who had been injured 1 year prior to the beginning of the study.

In the urodynamic examination, urination disorder due to thoracolumbar spinal cord injury is usually manifested with detrusor hyperreflexia, flabby bladder neck and proximal urethra, reduced bladder capacity, low compliance of the bladder, residue urine of varying amount and loss of coordination in both the detrusor and sphincter that result in low urine flow rate [24-26]. Urodynamic examination shows that maximum bladder capacity decreases, residue urine volume emerges or increases, maximum detrusor pressure increases, and maximum urinary flow rate decreases. After stem cell transplantation, urodynamic indicators showed some improvements: an increase in maximum bladder capacity and a decrease in maximum detrusor pressure were most significant (P = 0.09 and P = 0.023, respectively), maximum urinary flow rate also showed increasing tendency and residual urine volume decreased, indicating improvements in both urine storage and voiding function of the bladder. After stem cell treatment, changes in clinical manifestations of the patients were that urine could be sensed and retained for a period of time, voiding was smooth, voiding frequency decreased, and the amount of urine of each time increased and voiding time shortened. Only 2 of the 10 patients had urinary infection or urinary calculus (2/10, 20%). Patients of the rehabilitation group showed a significant decrease in maximum bladder capacity (P = 0.043). They also showed decreasing tendencies of maximum urinary flow rate, an increase in residual urine volume, and an increase in maximum detrusor pressure, but these changes were not statistically significant (P = 0.222, P = 0.082, and P = 0.374, respectively). These patients showed enuresis, frequent urination, and reduced urine volume of each voiding, indicating an abnormality in urine storage and the voiding function of the bladder. Nine of the 14 patients (64%) relied on daily intermittent catheterization and assisted urination through pressure on the lower abdomen. Due to repeated catheterization and excessive external force on squeezing the bladder, 8 of the 14 patients (57%) showed varying degrees of hematuria, urinary tract infections and urinary calculus, hydronephrosis in the renal pelvis and ureter, renal abnormalities, etc. The stem cell transplantation group showed significant improvements in voiding function compared with the rehabilitation group. They could better sense, retain, and void urine, and urinary tract infections and urinary calculus consequently decreased.

## Conclusions

This study demonstrated that umbilical cord mesenchymal stem cell transplantation is effective in the treatment for sequelae of thoracolumbar spinal cord injury. This method can alleviate lower limb muscle tension, increase limb strength, and improve urinating function. The method’s efficacy is more significant in comparison with rehabilitation therapy, and no adverse effects were found.

## Abbreviations

UCMSCs: Umbilical cord-derived mesenchymal stem cells
SC: Stem cell
SCI: Spinal cord injury
AIS: Abbreviated injury scale
ASIA: American spinal injury association
MEP: Motor evoked potentials

## References

[1] Li J, Liu G, Zheng Y, Hao C, Zhang Y, Wei B, Zhou H, Wang D (2011). The epidemiological survey of acute traumatic spinal cord injury (ATSCI) of 2002 in Beijing municipality. Spinal Cord.

[2] Reitz A (2012). Afferent pathways arising from the lower urinary tract after complete spinal cord injury or cauda equina lesion: clinical observations with neurophysiological implications. Urol Int.

[3] Zhang S, Wadhwa R, Haydel J, Toms J, Johnson K, Guthikonda B (2013). Spine and spinal cord trauma: diagnosis and management. Neurol Clin.

[4] Ibarra A, Martinon S (2009). Pharmacological approaches to induce neuroregeneration in spinal cord injury: an overview. Curr Drug Discov Technol.

[5] Kwon BK, Okon E, Hillyer J, Mann C, Baptiste D, Weaver LC, Fehlings MG, Tetzlaff W (2011). A systematic review of non-invasive pharmacologic neuroprotective treatments for acute spinal cord injury. J Neurotrauma.

[6] Ditunno JF, Cardenas DD, Formal C, Dalal K (2012). Advances in the rehabilitation management of acute spinal cord injury. Handb Clin Neurol.

[7] van Hedel HJ, Dietz V (2010). Rehabilitation of locomotion after spinal cord injury. Restor Neurol Neurosci.

[8] Beck KD, Nguyen HX, Galvan MD, Salazar DL, Woodruff TM, Anderson AJ (2010). Quantitative analysis of cellular inflammation after traumatic spinal cord injury: evidence for a multiphasic inflammatory response in the acute to chronic environment. Brain.

[9] Ghasemlou N, Bouhy D, Yang J, Lopez-Vales R, Haber M, Thuraisingam T, He G, Radzioch D, Ding A, David S (2010). Beneficial effects of secretory leukocyte protease inhibitor after spinal cord injury. Brain.

[10] Hernandez J, Torres-Espin A, Navarro X (2011). Adult stem cell transplants for spinal cord injury repair: current state in preclinical research. Curr Stem Cell Res Ther.

[11] Komuta Y, Teng X, Yanagisawa H, Sango K, Kawamura K, Kawano H (2010). Expression of transforming growth factor-beta receptors in meningeal fibroblasts of the injured mouse brain. Cell Mol Neurobiol.

[12] Lee SH, Chung YN, Kim YH, Kim YJ, Park JP, Kwon DK, Kwon OS, Heo JH, Ryu S, Kang HJ, Paek SH, Wang KC, Kim SU, Yoon BW (2009). Effects of human neural stem cell transplantation in canine spinal cord hemisection. Neurol Res.

[13] Park SS, Byeon YE, Ryu HH, Kang BJ, Kim Y, Kim WH, Kang KS, Han HJ, Kweon OK (2011). Comparison of canine umbilical cord blood-derived mesenchymal stem cell transplantation times: involvement of astrogliosis, inflammation, intracellular actin cytoskeleton pathways, and neurotrophin-3. Cell Transplant.

[14] Ra JC, Shin IS, Kim SH, Kang SK, Kang BC, Lee HY, Kim YJ, Jo JY, Yoon EJ, Choi HJ, Kwon E (2011). Safety of intravenous infusion of human adipose tissue-derived mesenchymal stem cells in animals and humans. Stem Cells Dev.

[15] Kaner T, Karadag T, Cirak B, Erken HA, Karabulut A, Kiroglu Y, Akkaya S, Acar F, Coskun E, Genc O, Colakoglu N (2010). The effects of human umbilical cord blood transplantation in rats with experimentally induced spinal cord injury. J Neurosurg Spine.

[16] Shang AJ, Hong SQ, Xu Q, Wang HY, Yang Y, Wang ZF, Xu BN, Jiang XD, Xu RX (2011). NT-3-secreting human umbilical cord mesenchymal stromal cell transplantation for the treatment of acute spinal cord injury in rats. Brain Res.

[17] Cao FJ, Feng SQ (2009). Human umbilical cord mesenchymal stem cells and the treatment of spinal cord injury. Chin Med J (Engl).

[18] Hu SL, Luo HS, Li JT, Xia YZ, Li L, Zhang LJ, Meng H, Cui GY, Chen Z, Wu N, Lin JK, Zhu G, Feng H (2010). Functional recovery in acute traumatic spinal cord injury after transplantation of human umbilical cord mesenchymal stem cells. Crit Care Med.

[19] Liao W, Xie J, Zhong J, Liu Y, Du L, Zhou B, Xu J, Liu P, Yang S, Wang J, Han Z, Han ZC (2009). Therapeutic effect of human umbilical cord multipotent mesenchymal stromal cells in a rat model of stroke. Transplantation.

[20] Malgieri A, Kantzari E, Patrizi MP, Gambardella S (2010). Bone marrow and umbilical cord blood human mesenchymal stem cells: state of the art. Int J Clin Exp Med.

[21] Zhang L, Zhang HT, Hong SQ, Ma X, Jiang XD, Xu RX (2009). Cografted Wharton's jelly cells-derived neurospheres and BDNF promote functional recovery after rat spinal cord transection. Neurochem Res.

[22] Dai GH, Liu XB, Zhang Z, Wang XD, Li M, Cheng HB, Hua RR, Shi J, Wang RZ, Qin C, Gao JH, An YH (2013). Comparative analysis of curative effect of CT-guided stem cell transplantation and open surgical transplantation for sequelae of spinal cord injury. J Transl Med.

[23] Putz C, Schuld C, Akbar M, Grieser T, Wiedenhofer B, Furstenberg CH, Gerner HJ, Rupp R (2011). Neurological and functional recovery in multiple injured patients with paraplegia: outcome after 1 year. J Trauma.

[24] de Groat WC, Yoshimura N (2012). Plasticity in reflex pathways to the lower urinary tract following spinal cord injury. Exp Neurol.

[25] Hadiji N, Miri I, Ben Salah FZ, Rahali H, Koubaa S, Lebib S, Dziri C (2009). [Neurogenic urinary bladder in patients with spinal cord injury. Protocol of surveillance and management]. Tunis Med.

[26] Chen Z, Sun S, Deng R, Cai D, Yuan X, Du G, Yang W, Ye Z (2009). The assessment of bladder and urethral function in spinal cord injury patients. J Huazhong Univ Sci Technolog Med Sci.

